# Rho GTPase activating protein 21-mediated regulation of prostate cancer associated 3 gene in prostate cancer cell

**DOI:** 10.1590/1414-431X2024e13190

**Published:** 2024-06-17

**Authors:** D.A. Alves, A.F. Neves, L. Vecchi, T.A. Souza, E.R. Vaz, S.T.S. Mota, N. Nicolau-Junior, L.R. Goulart, T.G. Araújo

**Affiliations:** 1 Laboratório de Genética e Biotecnologia, Instituto de Biotecnologia, Universidade Federal de Uberlândia, Patos de Minas, MG, Brasil; 2 Laboratório de Nanobiotechnologia Prof. Dr. Luiz Ricardo Goulart Filho, Instituto de Biotechnologia, Universidade Federal de Uberlândia, Uberlândia, MG, Brasil; 3 Laboratório de Biologia Molecular, Universidade Federal de Catalão, Catalão, GO, Brasil; 4 Laboratório de Modelagem Molecular, Instituto de Biotecnologia, Universidade Federal de Uberlândia, Uberlândia, MG, Brasil

**Keywords:** PCA3, ncRNA, Phage display, Prostate cancer

## Abstract

The overexpression of the prostate cancer antigen 3 (*PCA3*) gene is well-defined as a marker for prostate cancer (PCa) diagnosis. Although widely used in clinical research, *PCA3* molecular mechanisms remain unknown. Herein we used phage display technology to identify putative molecules that bind to the promoter region of *PCA3* gene and regulate its expression. The most frequent peptide PCA3p1 (80%) was similar to the Rho GTPase activating protein 21 (ARHGAP21) and its binding affinity was confirmed using Phage Bead ELISA. We showed that ARHGAP21 silencing in LNCaP prostate cancer cells decreased *PCA3* and androgen receptor (*AR*) transcriptional levels and increased prune homolog 2 (*PRUNE2*) coding gene expression, indicating effective involvement of ARHGAP21 in androgen-dependent tumor pathway. Chromatin immunoprecipitation assay confirmed the interaction between *PCA3* promoter region and ARHGAP21. This is the first study that described the role of ARHGAP21 in regulating the *PCA3* gene under the androgenic pathway, standing out as a new mechanism of gene regulatory control during prostatic oncogenesis.

## Introduction

Prostate cancer (PCa) accounts for 7.5% of all cancer cases and is the fifth leading cause of cancer death worldwide (1). Efficient screening and early detection are crucial for the identification of the tumor at a curable stage, which allows successful treatment and minimizes complications associated with refractory and metastatic cases (2).

Prostate cancer associated 3 (*PCA3*) gene, first named as *DD3*, is a prostate tumor-specific biomarker with high transcriptional levels detected in the blood, biopsies, and urine (3,4). *PCA3* score (Progensa PCA3) was approved by the Food and Drug Administration in 2012 and measures the concentration of *PCA3* and *PSA* RNA in the urine collected after digital rectal examination (DRE), guiding further biopsies (5). Moreover, the usefulness of measuring *PCA3* in blood samples has been demonstrated for the early detection of PCa and for monitoring circulating PCa cells (6-8). It remains one of the most evaluated and investigated biomarkers for PCa diagnosis and prognosis assessment (4,7).

*PCA3*, first identified by Bussemakers et al. (3), is located in chromosome 9 at position q21.2, has 23,112 nucleotides including four exons (NCBI: #NC_000009), which are not translated, defining it as a long non-coding RNA (lncRNA). More recent data show that the *PCA3* gene structure is a complex transcriptional unit, with alternative splicing, polyadenylation, and different isoforms (9). Prostatic tumors are directly correlated with the 277-bp amplicon containing exon 2 splicing fragment (10). Moreover, *PCA3* was mapped in an antisense orientation of the prune homolog 2 (*PRUNE2*) coding gene within its intron 6, as a negative transdominant oncogenic molecule (11).

The promoter region of *PCA3* has 500 nucleotides (12). Polymorphisms within this region have been linked to the tumorigenic process and the absence of a TATA box element has been associated with promiscuous transcriptional initiation (13). Although the role of *PCA3* in controlling the expression of androgen-responsive and cancer-related genes, such as epithelial-mesenchymal transition markers, has been previously described (14,15), the inherent biological function of *PCA3* has remained elusive. Therefore, the identification of regulatory proteins that may either activate or inhibit the development of PCa, particularly through *PCA3* regulation, has great importance, especially in understanding and reverting the malignant phenotypes.

Herein, we used phage display technology to identify putative molecules that bind to the promoter region of *PCA3* gene and regulate its expression. We selected short peptides binding to the promoter region of *PCA3*; one of them was highly frequent (80%) and similar to the RhoGAP Rho GTPase activating protein 21 (ARHGAP21/ARHGAP10). Therefore, this work defined a biologic functional action of ARHGAP21 as a regulator of *PCA3* in androgen-sensitive cells paving a way for new translational strategies in PCa management.

## Material and Methods

### Patient samples and nucleic acid extraction

This study was conducted in the Prof. Dr. Luiz Ricardo Goulart Filho Nanotechnology Laboratory of the Universidade Federal de Uberlândia (UFU) upon approval by the Ethics Committee in Research (CEP 005/2001). All subjects enrolled in this study signed an informed consent form. The recruitment of participants was carried out by the urology service of the Clinical Hospital of UFU, which evaluated urinary tract symptoms, prostate-specific antigen (PSA) serum levels, and DRE. None of the patients underwent treatment before surgery. For initial *PCA3* screening, 26 peripheral blood samples were collected and grouped into two classes according to histopathological results: 24 with PCa and 2 cases with benign prostatic hyperplasia (BPH).

Total RNA and DNA were extracted from samples according to previously published protocols (16,17). DNA and RNA yield and A260/280 ratio were monitored using a NanoDropND-100 spectrometer (NanoDrop Technologies, USA).

### *PCA3* detection and promoter region amplification

Reverse transcription was carried out following the protocol published by Neves et al. (7). β2-microglobulin (*B2M*) gene amplification was conducted to confirm the success of cDNA synthesis, and the nested-PCR reaction was performed for the detection of *PCA3* transcripts and selection of positive patients. Either the reactions or primer sequences were previously described (7).

Specific primers were designed for promoter segment amplification: 5′-CACTAGAGGAGCACCTTAGGAATTG-3′ and 5′-CATATTCTGAAGTCAGAGTGTTCCAG-3′. DNA from positive patients for *PCA3* transcripts was extracted and used in a PCR with forward biotinylated primer. The amplification was performed with 1X PCR buffer (20 mM Tris-HCl - pH 8.0, 0.1 mM EDTA, 1 mM DTT, glycerol 50% v/v) (Invitrogen, USA), 200 µM of dNTPs (Invitrogen), 0.25 µM of each primer (Invitrogen), 1 U of Platinum^®^ Taq DNA polymerase (Invitrogen), 3 mM of MgCl_2_ (Invitrogen), 2 µL of DNA, and milli-Q water for a final volume of 20 µL. The reaction was incubated at 95°C/1 min followed by 34 cycles at 94°C/40 s, 59°C/50 s, and 72°C/40 s with a final extension of 72°C/10 min. Amplicons were analyzed by electrophoresis in 1.5% agarose gel stained with GelRed 1X (Biotium, USA).

The amplified 460 bp fragment, corresponding to the promoter segment of the *PCA3* (*PCA3*prom), was purified by DNA precipitation with ammonium acetate (7.5 M) and the pellet was resuspended in 10 μL of water. DNA concentration and quality were checked using the spectrophotometric absorbance readings at 260 and 280 nm.

### Phage display

For the selection of binding peptides to the *PCA3*prom, we used a PhD-12mer phage display peptide library kit (New England Biolabs, USA) fused to the pIII protein of M13 bacteriophage. A sample of the library containing 1×10^11^ phage particles was submitted to three cycles of selection and amplification.

PCR amplification of the promoter region with a specific biotinylated primer was batch-purified on streptavidin-coated Dynabeads (Invitrogen). A fresh aliquot of 300 μL streptavidin beads was washed 3 times with SSC Buffer (3 M NaCl and 0.3 M sodium citrate). After the last wash, 200 pmoles of biotinylated product, diluted in an equal volume of binding buffer (1:52 mM Tris, 0.1 M NaCl, 4.2 mM MgCl_2_, 4.6 mM KCl, 2.7 mM CaCl_2_, 2% Tween 20), were added to the beads and incubated for 30 min at 36°C under gentle shaking. Beads were captured by a magnetic platform to remove the supernatant and the bead/DNA complex was washed with 1 mL of wash buffer pH 8.0 (0.1 M Tris, 2.5 M NaCl, 0.3 M sodium azide). The bead/DNA complex was incubated with the phage library for 1 h at 37°C under agitation. The unbound phages were discarded by washing five times with 500 µL of binding buffer. Phages that were bound to *PCA3*prom were eluted with 0.2 M glycine pH 2.2 and neutralized with 1 M Tris-HCl pH 9.1. The eluted phages were amplified in *Escherichia coli* strain ER2738 (New England Biolabs), and viral particles were precipitated with PEG-8000/NaCl. The amplified phages were then used in the next round of selection. Three cycles of selection were performed, and titration was conducted as described elsewhere (18).

### Viral DNA extraction and sequencing

Phages from the third round were isolated and transferred to deep-well plates containing 1.2 mL of ER2738 culture in the exponential growth phase (absorbance 600 nm ∼ 0.3) for DNA extraction. Phage particles were isolated from bacteria through centrifugation at 3,500 *g* for 10 min, at room temperature. The supernatant was transferred to a new plate and precipitation was performed with 1/6 volume PEG/NaCl (20% w/w, polyethylene glycol 8000) and iodide buffer (10 mM Tris-HCl (pH 8.0), 1 mM EDTA, and 4 M NaI). Phage DNA was precipitated with absolute ethanol, followed by a wash with 70% ethanol, and resuspended in 20 μL Milli-Q water.

The quality of the single-stranded DNA was verified by electrophoresis in 0.8% agarose gel stained with GelRed 1X. The sequencing reaction was performed in the ABI 3500 (Applied Biosystems, USA) platform using the primer 96M13 (5′-CCCTCATTAGTTAGCGCGTAACG-3′), following the manufacturer's instructions.

### Identification of selected peptides

Corresponding 12-mer peptide sequences were deducted using the ExPASy Proteomics and Sequence Analysis tool (http://web.expasy.org/translate/). Local alignment by the BLASTP™ tool (http://blast.ncbi.nlm.nih.gov/Blast.cgi?PAGE=Proteins) identified similar targets in Homo sapiens protein database, confronted with the UniProtKB. EMBOSS-Needle (http://www.ebi.ac.uk/Tools/psa/emboss_needle/) was also used to confirm blast alignment between the peptides and proteins that could be representative in blast. For sequence similarity analysis, multiple sequence alignment was performed by Clustal Omega (http://www.ebi.ac.uk/Tools/msa/clustalo/). In order to identify potential false positives, selected peptides were analyzed through PepBank (http://pepbank.mgh.harvard.edu/) and SAROTUP (http://i.uestc.edu.cn/sarotup3) searches for peptides binding to unintended materials.

### Cell culture

The tumorigenic prostatic cell lines LNCaP (CRL-1740; androgen-sensitive) and PC-3 (CRL-3471; castration-resistant) and the nontumorigenic lineage RWPE-1 (CRL-11609) were obtained from ATCC and cultured according to the supplier's recommendations. Flasks and plates were incubated at 37°C and 5% CO_2_ until 80% confluence free from mycoplasma and authenticated through STR (short tandem repeat) analysis.

### RNA extraction and qPCR assays

Total RNA was extracted from prostate cells using TRIzol Reagent^®^ (Invitrogen), according to the manufacturer's recommendations. The quality of RNA was assessed by agarose gel electrophoresis and was quantified spectrophotometrically using the NanoDropND-100. Next, as described above, 1.0 µg total RNA was used for reverse transcription (RT).

qPCR was performed using an ABI 7300 Real-Time PCR Systems (Applied Biosystems). Reactions were prepared for a final volume of 10 μL with 1.0 μL of cDNA and 5.0 µL of Power Master Mix SYBR^®^ Green reagent (Applied Biosystems) and incubated at 95°C for 5 min, followed by 40 cycles of 15 s at 94°C, 30 s at annealing temperature, and 30 s at 72°C. Primer sequences and annealing temperatures are described in Table 1. *B2M* was used as an internal control as previously published (19). The average relative quantification cycle for each gene was normalized against *B2M* and ΔCt was calculated.

**Table 1:** Oligonucleotide sequences and annealing temperature used for qPCR assays.

Target (reference)	Sequence 5′-3′(forward/reverse)	Annealing temperature (°C)	Amplicon
*B2M*	CCTGCCGTGTGAACCATG GCGGCATCTTCAAACCTC	60°C	93 bp
*PCA3* (10)	CGAGGGAGACCAGGAAGAT ATCGATGACCCAAGATGGCG	62°C	277 bp
*ARHGAP21	ACCGATAAGGGCAAGCGAG CTCTTCCTCAGACGGAGTCGTC	60°C	120 bp
*AR* (10)	GTGTAAGTTGCGGAAGCCAGG CATGTGGAAGCTGCAAGGTCT	64°C	516 bp
*PRUNE2	CAGCCCTTCTGCATGGAAC TTTTCTCTTGGGTAGGTCTGGG	60°C	172 bp

* Set of primers designed and described for the first time in this research. *B2M*: Beta-2-Microglobulin; *PCA3*: prostate cancer associated 3; *ARHGAP21*: Rho GTPase activating protein 21; *AR*: androgen receptor; *PRUNE2:* prune homolog 2.

### Chromatin immunoprecipitation (Chip)

In order to confirm the interaction between the *PCA3* promoter region and ARHGAP21, protein-DNA from LNCaP cells was crosslinked through antibody specificity. Trypsinized LNCaP cells were washed with PBS 1X followed by incubation with 1% formaldehyde for 30 min, at room temperature. Subsequently, 125 mM glycine was added for 5 min at room temperature and then washed three times with PBS 1X through centrifugation at 90 *g* (5 min, room temperature). The pellet was resuspended in 750 µL of lysis buffer (125 mM TRIS-Cl; 0.1 M NaCl; 0.1% SDS; 0.1% Tween-20; 1% Triton X-100) and incubated for 10 min on ice. Sonication with two pulses of 10 s, with a 30 s interval on ice, was performed for the fragmentation of chromatin. Cell lysate (input) was reserved to confirm the detection of *PCA3*prom after sonication procedures.

For antibody-bead crosslink, 50 µL of protein G magnetic beads (Dynabeads^®^ Protein G, Thermo Fisher Scientific, USA) were used. Beads were washed with 0.01% PBS-T and incorporated with anti-ARHGAP21 (1:50, HPA036610; Sigma-Aldrich, USA) and anti-EGFR (1:100, PA1−1110; Thermo Fisher Scientific); all antibodies were diluted in PBS 1X. Chip with anti-EGFR was performed as negative control with unrelated antibody. The bead-antibody solution was incubated for 20 min at room temperature under agitation. The bead-antibody crosslink, via imidoester and amine reactions, was performed using 50 mM DMP (dimethyl pimelimidate dihydrochloride; Sigma) in 0.2 M triethanolamine, pH 8.2 for 1 h. The reaction was stopped by washing the bead-antibody complex with 50 mM glycine followed by another 0.05% PBS Tween-20 wash.

The immunoprecipitation was carried out by incubating 50 µL of antibody beads with the LNCaP cell lysate for 1 h at room temperature. Bead-antibody-protein-DNA complexes were separated on magnetic column and eluted with an elution buffer (100 mM NaHCO_3_; 1% SDS), treated with proteinase-k for 1 h at 60°C. The DNA was purified from the eluted solution using the Realiprep^TM^ (Promega, USA) column following the manufacturer's instructions. Electrophoresis was carried out on 1.5% agarose gel stained with GelRed 1X. DNA from LNCaP cells grown in culture medium was used as positive control for the amplification of *PCA3*prom. Chip assay without any cellular material (blank) was also performed. Primers designed for nitric oxide synthase 3 (*NOS3*) gene (5′-ATGCTGCCACCAGGGCATCA-3′ and 3′-GTCCTTGACTCTGACATTAGGG-5′) were used as a link-specificity control.

### Gene silencing

Silencing was carried out using a set of 19 endoribonucleases prepared as small interfering RNAs (Mission^®^ esiRNA, Sigma) targeting the human *ARHGAP21* gene (esiARHGAP21) and the negative control (Mission^®^ esiGFP, Sigma) targeting the green fluorescent protein. LNCaP cells were grown in 6-well plates and maintained in RPMI-supplemented medium with 10% FBS until 70% confluence. On the day before transfection, the medium was used without antibiotics. Transfections were performed with 400 ng of siRNAs using Lipofectamine 3000^®^ (Invitrogen) vesicles according to the supplier's instructions. Twenty-four hours after transfection, 1 mL of complete medium was added, and cells were trypsinized after 48 h of transfection for further analyses.

### Western blot analysis

Total proteins from 1×10^6^ esiRNA-transfected LNCaP cells, as described earlier, were extracted using NE-PER™ Nuclear and Cytoplasmic Extraction Reagents (Thermo Fisher Scientific) according to the manufacturer's instructions. BCA assay (Pierce™ BCA Protein Assay Kit; 23225; Thermo Fisher Scientific) was used for protein quantification and standardization. The protein extracts were separated using 10% SDS-PAGE and transferred to a nitrocellulose membrane (Hybond, GE Healthcare, USA). The membrane was blocked for 1 h with PBS in 5% milk and then incubated with anti-ARHGAP21 (1:600; 22183-1-AP; Proteintech^TM^, USA). Anti-Lamin B2 (1:1000, SAB2702205, Sigma) was used as a loading control. The goat anti-rabbit IgG HRP (1:3000; G21234; Thermo Fisher Scientific) was used as a secondary antibody. The subsequent washing steps and detection procedures were performed according to the ECL Plus manual (GE Healthcare).

### Phage PCA3p1 purification and Bead ELISA

The highest frequent clone, PCA3p1, was used in a Bead ELISA assay to verify its reactivity with *PCA3*prom. PCA3p1 and a wild-type phage clone, which does not express foreign exogenous peptides, were amplified using early-log *E*. *coli* ER2738 as a host and purified by adding one-sixth volume of 20% polyethylene glycol-8000 and 2.5 M NaCl overnight at 4°C. The precipitate was resuspended in 200 µL of PBS (phosphate-buffered saline).

For the Phage Bead-ELISA assay, 200 pmol of biotinylated *PCA3*prom, prepared as described above, were incubated with 3×10^5^ U streptavidin-beads (Dynabeads™ MyOne™ Streptavidin T1; #65601; Thermo Fisher Scientific) for 30 min at 36°C in 260 µL of binding buffer under agitation. After magnetic separation, the bead-*PCA3*prom was washed three times with wash buffer and 1×10^10^ plaque-forming unit (pfu) of the selected clone PCA3p1 and wild-type phage diluted in PBS1x were added and incubated for 1 h, 37°C, under slight agitation. The solution was washed 3 times with PBS-T (0.05%) and anti-M13HRP (horseradish peroxidase; 11973-MM05T-H; Sino Biological^TM^, China) (1:5000) secondary antibody was incubated for 1 h at 37°C. After magnetic separation, the reaction was revealed with OPD (ortho-phenylenediamine) SigmaFast™ (Sigma-Aldrich) and read at 450 nm on Multiskan GO^®^ (Thermo Fisher Scientific).

### Modulatory effects of PCA3p1 phage in LNCaP cells

The transcriptional levels of *PCA3*, *PRUNE2*, *ARHGAP21*, and *AR* were quantified after stimulation of LNCaP cells with the PCA3p1 phage. According to the proliferation assay, 10^7^ pfu of PCA3p1 were used to treat 7×10^5^ cells for 24 and 48 h. Total RNAs were extracted using the TRIzolReagent^®^ followed by RT-qPCR assay as described above.

### *In silico* predictions

The three-dimensional structure of the isoform consensus sequence (710-1860aa) of ARHGAP21 (NCBI: NP_001354377.1) was modeled using the online server I-TASSER (20). The *PCA3*prom (1-460bp) (NCBI: AF279290.1) DNA structure was predicted using the 3DDART software and PCA3p1 peptide (HGPNIDHLATLH) was modeled using the PEP-FOLD3 software (21). Structural analyses and validations were carried out based on the software internal scores and online verification tools, such as SAVES v5.0 (22) and RAMPAGE: Ramachandram Plot Assessment (23), which together evaluate the stereochemistry and spatial coherence, compatibility between the linear sequence (1D), and the predicted model (3D). PATCHDOCK software (https://bioinfo3d.cs.tau.ac.il/PatchDock/) was used to perform both PCA3p1-*PCA3*prom and ARHGAP21-*PCA3*prom docking to simulate and visualize possible interactions between the peptide and protein to the promoter region of the *PCA3* gene. The PyMOL Molecular Graphics System Software Version 2.3.0 Schrödinger LLC (https://pymol.org/) was used to view, analyze, edit, and export the images.

### Statistical analysis

One-way analysis of variance (ANOVA) was used to verify differences between the means of independent groups whose data were in normal distribution. Unpaired *t*-test was used to assess the differences between the means of two groups. All analyses were performed using GraphPad Prism software 7.0 (USA). P<0.05 was considered to be statistically significant.

## Results

### *PCA3* promoter amplicon as a target for phage display

**Figure 1:**
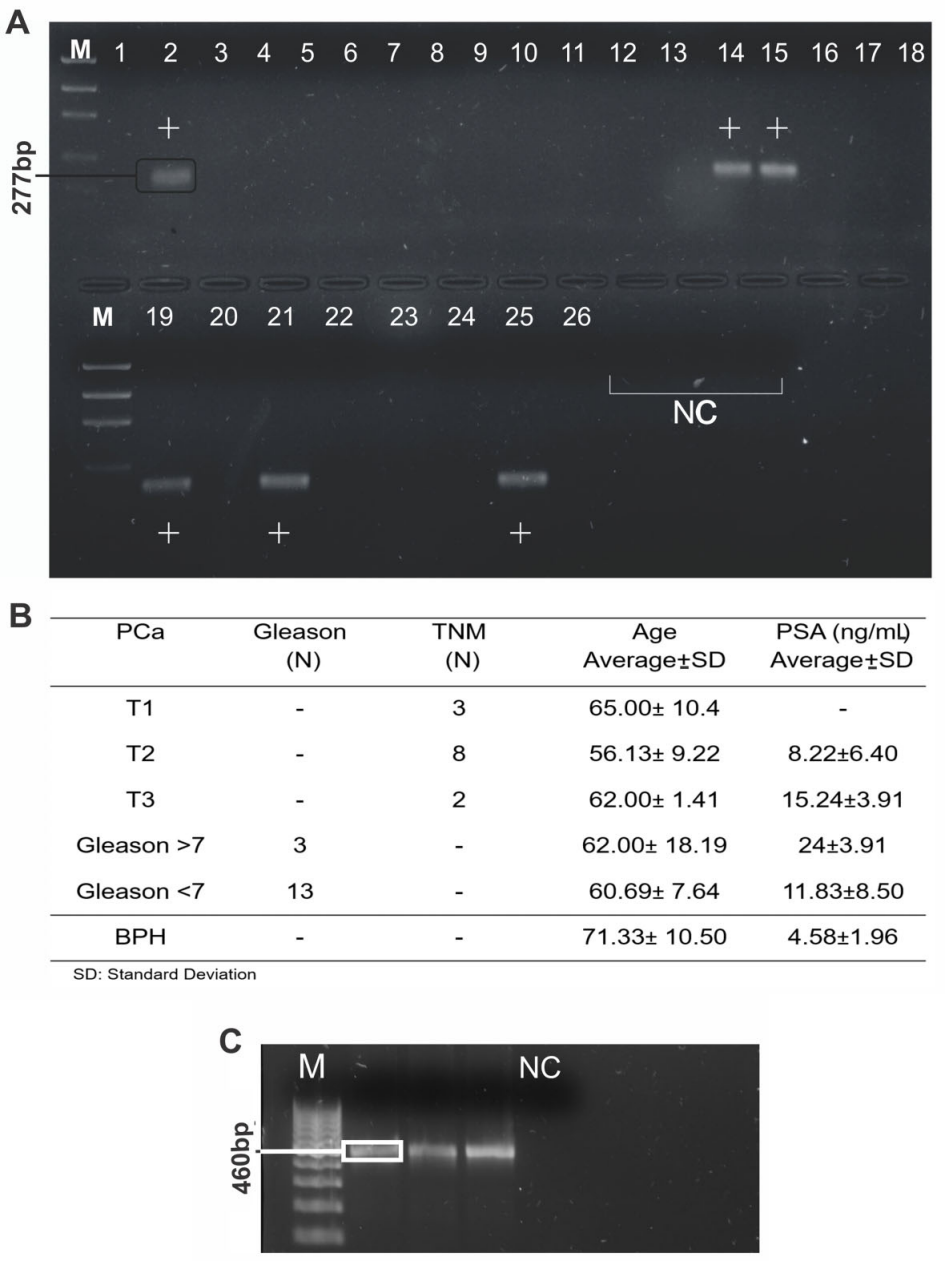
Prostate cancer associated 3 (*PCA3*) amplification in peripheral blood samples. **A**, Representation of qualitative RT-Nested PCR of *PCA3* gene. **B**, Clinicopathological data of selected patients (n=26 patients); 24 PCa (1-17, 10-21, and 23-26) and 2 BPH (18 and 22). **C**, Amplification of *PCA3* promoter segment with the biotinylated sense primer. M: 100-pb ladder; NC: negative control. PCa: prostate cancer; BPH: benign prostatic hyperplasia; TNM: tumor, node, metastasis; PSA: prostate-specific antigen.

*PCA3* remains as one of the most specific PCa biomarkers (4) and its detection in peripheral blood demonstrated its usefulness in PCa diagnosis and in monitoring PCa circulating cells (6,7). Based on this, we performed RT-Nested PCR assays in order to identify its transcripts in peripheral blood samples of 26 patients. The detection of a 277-bp fragment from the splicing of exon 2 of *PCA3* and clinicopathological characteristics of selected patients are shown in Figure 1A and B. This is the classical isoform amplified in PCa samples (3,10) and we detected this transcript in six men (25%), all of them diagnosed with PCa. No amplification was observed in BPH samples. DNA was extracted from the peripheral blood of *PCA3-*positive patients, and the 460-bp promoter segment flanked by biotinylated (Figure 1C) primers was amplified.

### Peptides selected as putative ligands to *PCA3* promoter

**Figure 2:**
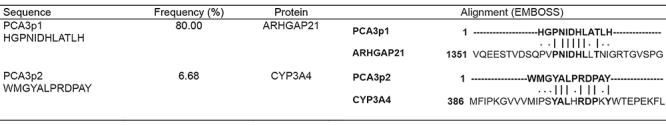
Sequence and alignment of PCA3p1 and PCA3p2. ARHGAP21: Rho GTPase activating protein 21; CYP3A4: Cytochrome P450 3A4.

A total of 30 clones were selected against the *PCA3*prom amplicon. After sequencing, different peptides were obtained after the third round of selection. PCA3p1, with a putative sequence HGPNIDHLATLH, represented 80% of selected peptides. PCA3p2, composed by WMGYALPRDPAY, repeated two times. The PCA3p1 peptide aligned with Rho GTPase activating protein 21 (ARHGAP21) with 88% of identity, and PCA3p2 showed 78% of identity with Cytochrome P450 3A4 (CYP3A4) (Figure 2).

### ARHGAP21 regulated *PCA3* transcripts

**Figure 3:**
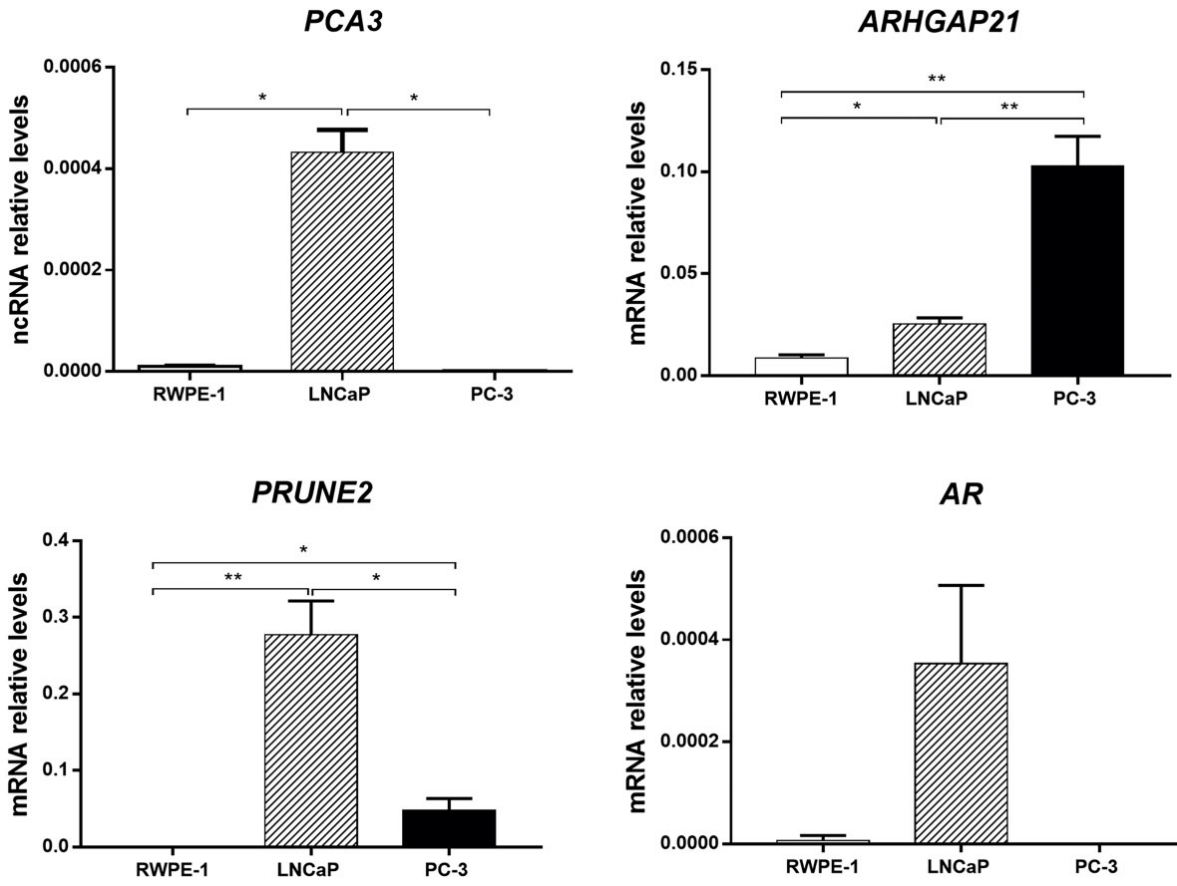
Basal transcriptional levels of prostate cancer associated 3 (*PCA3*), Rho GTPase activating protein 21 (*ARHGAP21*), prune homolog 2 (*PRUNE2*), and androgen receptor (*AR*) in RWPE-1, LNCaP, and PC-3 cells. *PCA3* transcripts were upregulated in LNCaP cells, the best model to study this lncRNA pathway. *P<0.05, **P<0.01 (ANOVA).

Since PCA3p1 is similar to the protein ARHGAP21, we sought to describe whether the endogenous ARHGAP21 could indeed bind to the *PCA3*prom. In order to do so, we quantified the expression of *PCA3* transcripts in the prostate cell lines. We analyzed the transcriptional levels of two *PCA3*-related genes, *PRUNE2* and *AR*, and the mRNA expression of *ARHGAP21* in all lineages (Figure 3). Higher *PCA3* transcripts were detected in LNCaP cells with low amplification in PC-3, as previously described (14). *AR* and *PRUNE2* were also upregulated in LNCaP cells, whereas *ARHGAP21* was highly expressed in PC-3 cells. Based on these results, we decided that LNCaP cell line represented the best model to describe the *PCA3* pathway.

**Figure 4:**
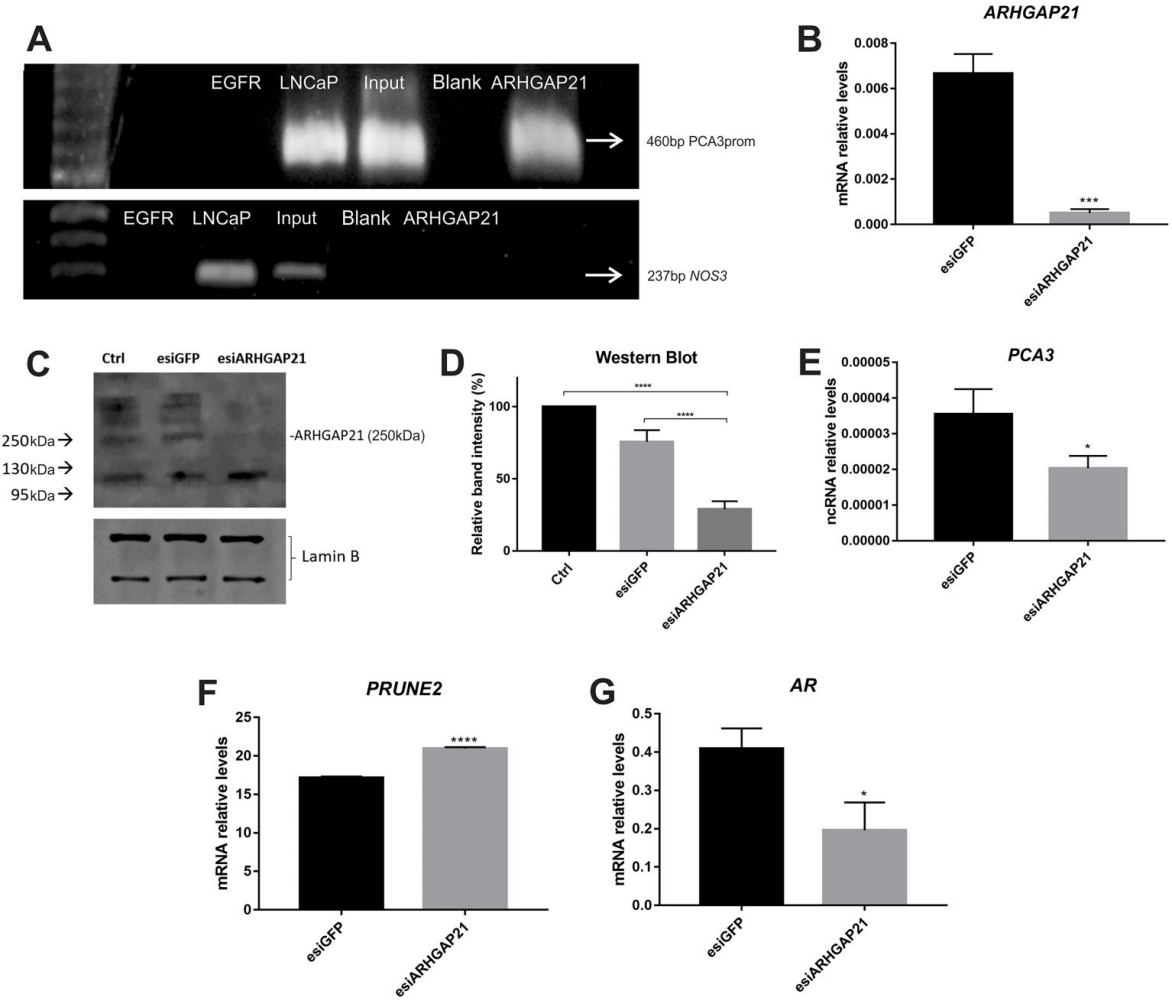
Rho GTPase activating protein 21 (ARHGAP21) binds to *PCA3* promoter region (*PCA3*prom) and regulates its transcripts. **A**, Chromatin immunoprecipitation (Chip) and iRNA assays were performed to confirm the regulation of prostate cancer associated 3 (*PCA3*) mediated by endogenous ARHGAP21. Chip was carried out with anti-ARHGAP21 (ARHGAP21) or anti-EGFR antibody (EGFR) followed by *PCA3* promoter and nitric oxide synthase 3 (NOS3) amplification. DNA extracted from LNCaP cells (LNCaP) and LNCaP cell lysate (input) were used as controls. Chip assay was also performed in the absence of cellular material (Blank). **B**, RT-qPCR quantification of LNCaP cells silenced for *ARHGAP21* (esiARHGAP21) compared to cells subjected to silencing of the irrelevant gene, *GFP* (esiGFP). **C**, Western blotting analysis of ARHGAP21 (250 kDa) expression in LNCaP cells. The image shows the *ARHGAP21* silencing (esiARHGAP21) compared to the untreated cells (Crtl) and the cells subjected to silencing of the irrelevant gene, *GFP* (esiGFP). Lamin B was used as a loading control of protein extracts. **D**, Quantification of western blotting image by ImageJ software (USA). Three independent stainings were performed. **E**, Transcriptional levels of *PCA3* in the LNCaP cells silenced for *ARHGAP21* (esiARHGAP21) compared to the cells subjected to silencing of the irrelevant gene, *GFP* (esiGFP). **F**, mRNA relative expression levels of *PRUNE2* and (**G**) *AR* were recorded in LNCaP-*ARHGAP21* silenced cells. bp: base pairs; kDa: kilodaltons. Data are reported as means±SD. *P<0.05, ***P<0.001, ****P<0.0001 (Student's *t*-test and ANOVA).

We performed a Chip assay to confirm the binding of endogenous ARHGAP21 to the promoter region of *PCA3*. The precipitation was carried out using an anti-ARHGAP21 antibody, and the presence of ARHGAP21-*PCA3*prom complex in the immunoprecipitated fraction was confirmed by a PCR reaction with specific primers for the *PCA3*prom region. As positive controls, we amplified the *PCA3*prom starting both from the DNA extracted from LNCaP cells and from a non-immunoprecipitated cell lysate (input). As negative controls, we performed a Chip assay without any cellular material (blank), and a Chip reaction was performed by using an anti-EGFR antibody as non-specific antibody. As shown in Figure 4A, the 460-bp segment of the *PCA3*prom was amplified in cell lysates immunoprecipitated with anti-ARHGAP21 as well as in the positive control and the input sample. In samples immunoprecipitated with anti-EGFR (negative control), the *PCA3*prom was not amplified, confirming the ability of the anti-ARHGAP21 in capturing the ARHGAP21-*PCA3*prom complex. To verify whether the DNA binding by ARHGAP21 was gene-specific, primers for *NOS3* gene were used. The 237-bp fragment was amplified on LNCaP and “Input” samples, while there was no amplification when precipitation with anti-ARHGAP21 was performed.

In order to demonstrate that endogenous ARHGAP21 indeed regulated *PCA3* transcripts, we silenced ARHAGAP21 in LNCaP cells (esiARHGAP21) and compared to LNCaP cells in which an irrelevant gene (*GFP*) was knocked down (esiGFP). We succeeded in silencing ARHGAP21 by showing a decrease of 92.4% of *ARHGAP21* transcriptional levels (Figure 4B). We also quantified with the ImageJ software the ARHGAP21 protein levels visualized by western blotting, and esiARHGAP cells showed a 62% of protein reduction compared to control cells (untreated cells - control) and, compared to esiGFP cells, a 52.2% reduction was achieved (Figure 4C and D).

Thereafter, we demonstrated that a reduction of ARHGAP21 expression led to a significant decrease of *PCA3* expression. We speculated that, by binding to *PCA3*prom region, ARHGAP21 regulated the expression of *PCA3* (Figure 4E). In addition, in LNCaP-ARHGAP21 silenced cells, there was an increase in *PRUNE2* expression and a decrease in *AR* expression (Figure 4F and G).

### Effects of PCA3p1 on PCa cells

**Figure 5:**
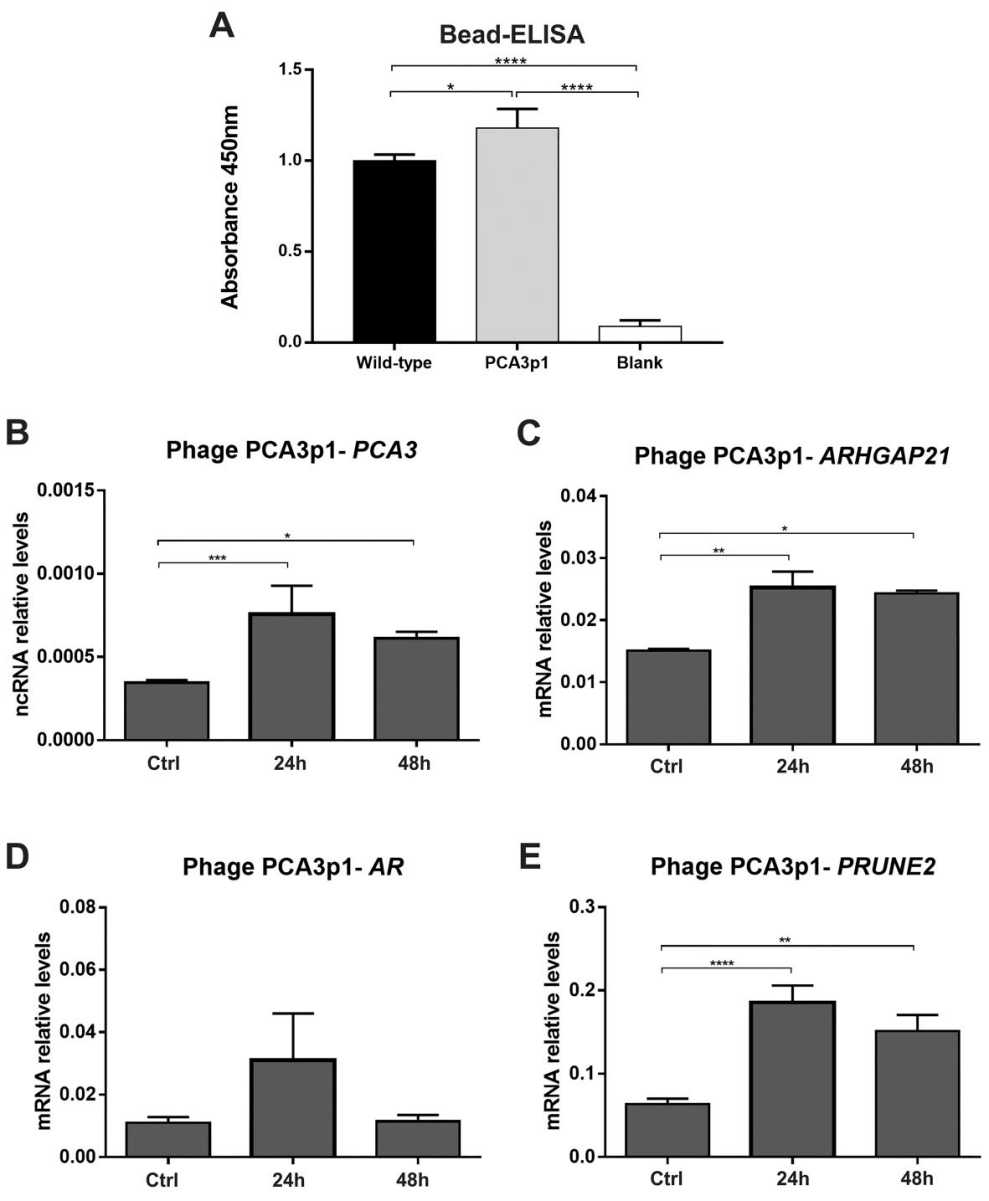
Phage binding analysis. **A**, Bead-ELISA performed with a biotinylated *PCA3* promoter amplicon and streptavidin-conjugated magnetic beads. The assay was carried out with PCA3p1 phage (PCA3p1) and wild-type phage that do not display any peptide (wild-type). Wells without phage particles (blank) were included as control. qPCR assays were also performed after the treatment of prostate cells with PCA3p1. **B**, Transcriptional levels of *PCA3* (prostate cancer associated 3), (**C**) *ARHGAP21* (Rho GTPase activating protein 21), (**D**) *AR* (androgen receptor), and (**E**) *PRUNE2* (prune homolog 2) genes quantified upon cell stimuli with 10^7^ phage particles of PCA3p1 for 48 h. Data are reported as means±SD. *P<0.05, **P<0.01, ***P<0.001, ****P<0.0001 (ANOVA).

In order to validate the binding specificity of peptide PCA3p1, expressed on the pIII protein of M13 phage, a Bead-ELISA assay was performed using the biotinylated *PCA3*prom and magnetic beads conjugated to streptavidin (Figure 5A). As control, the reaction was carried out in the absence of phages (blank), and the reactivity of PCA3p1 phage was compared to the wild-type phage. PCA3p1 phage showed a higher reactivity (P=0.0489) and, due to the absence of displayed peptides on the surface of wild-type phage, we speculated that the increased reactivity of PCA3p1 phage was due to the specific binding activity of PCA3p1 peptide to *PCA3*prom sequence.

We next decided to analyze the effect of the PCA3p1 on the regulation of *PCA3* expression in LNCaP cells. We therefore treated LNCaP cells with the wild-type phage as control and compared with the cells treated with the PCA3p1 phage through RT-qPCR analysis (Figure 5B-E). We showed that, indeed, in LNCaP cells, a significant (P<0.05) increase of 119.48% was verified upon 24 h and 77.15% upon 48 h in *PCA3* expression after treatment with PCA3p1 (Figure 5B). Regarding the transcriptional levels of *ARHGAP21* (Figure 5C), the levels also increased after treatment with PCA3p1 phage. There was no effect on expression of *AR* (Figure 5D), and *PRUNE2* transcripts increased in 191.78% after 24 h and 137.4% after 48 h of treatment with the PCA3p1 phage (Figure 5E).

### Molecular docking and structural alignment simulations

**Figure 6:**
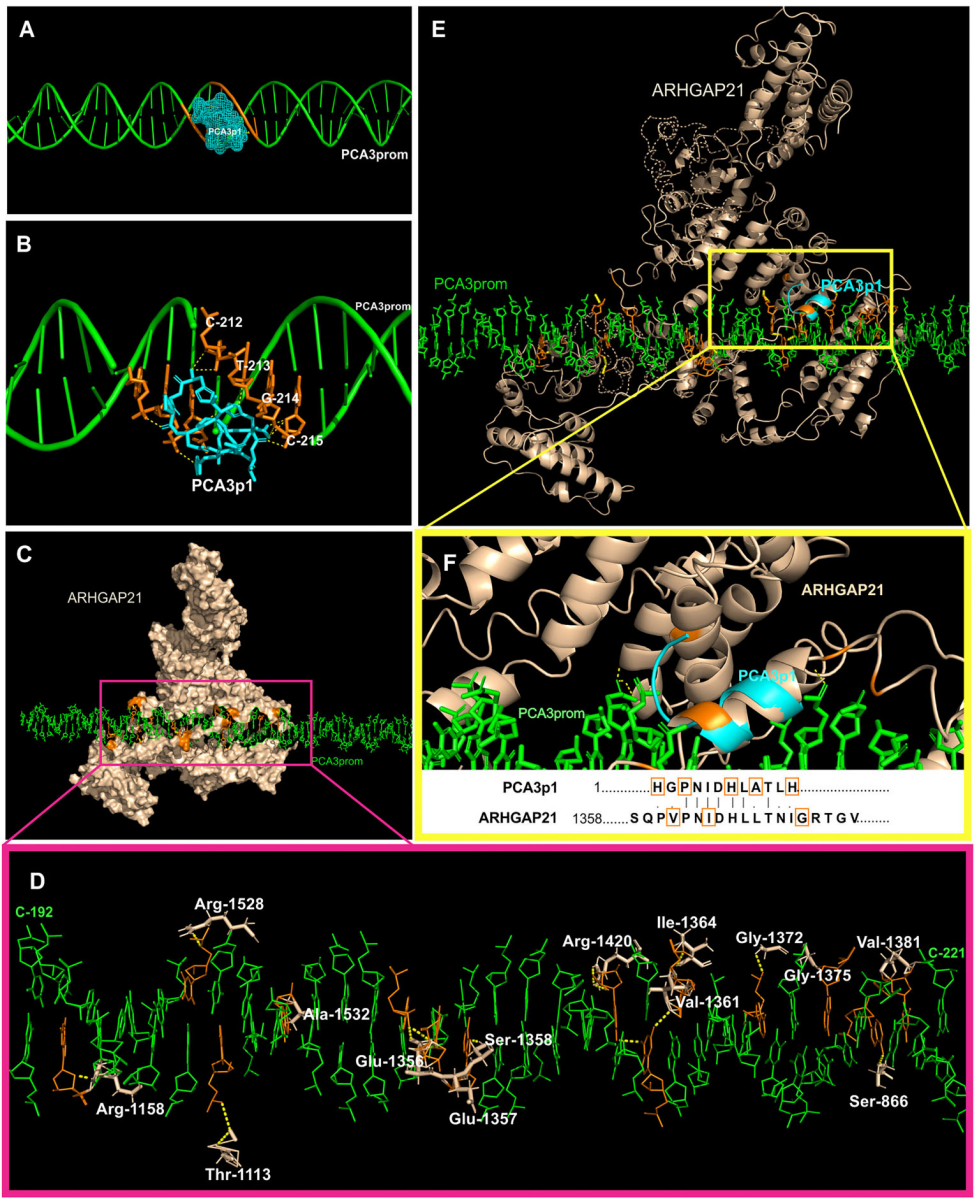
*In silico* predictions of the interaction between the ARHGAP21 protein and the peptide PCA3p1 to the promoter region of the *PCA3* gene. **A**, Overview of the peptide PCA3p1 (HGPNIDHLATLH) (cyan), expressed on the surface of the M13 phage and the *PCA3*prom (green). **B**, Zoom of the binding interaction between PCA3p1 and *PCA3* promoter region. **C**, Docking overview between the ARHGAP21 (wheat) and *PCA3*prom. **D**, Identification of ARHGAP21 binding residues (wheat) and interaction sites onto *PCA3*prom. **E**, Structural alignment between PCA3p1 and ARHGAP21. **F**, Enlarged image of interaction sites between the PCA3p1 peptide and ARHGAP21 with DNA and homology between the binding sites. Polar contacts are shown as dashed yellow lines, and the interaction residues are marked in orange.

Based on the fact that the experiments pointed to the interaction between the ARHGAP21 protein and the promoter region of the *PCA3* gene (GenBank: AF279290.1) (24), we performed an *in silico* prediction simulating how this interaction would probably happen to identify the most physicochemically favorable interaction site between PCA3p1 (HGPNIDHLATLH) and the *PCA3*prom amplicon (460-bp). The results are shown in Figure 6.

Figure 6A shows the docking between peptide PCA3p1 and *PCA3*prom. The interaction occurred in the minor groove of the double helix, favoring a greater contact. In Figure 6B, the polar bonds formed between the *PCA3*prom and the peptide are demonstrated. The nucleotides (forward strand) of the promoter sequence that interact with the PCA3p1 peptide are cytosine-212 (C-212), thymine-213 (T-213), guanine-214 (G-214), and cytosine-215 (C-215).

When analyzing the ARHGAP21-*PCA3*prom complex (Figure 6C), the protein conforms to create a DNA binding pocket, binding to both 5′-3′ and 3′-5′ strand in multiple sites, such as Ser-866, Thr-1113, Arg-1158, Glu-1356, Glu-1357, Ser-1358, Val-1361, Ile-1364, Gly-1372, Gly-1375, Val-1381, Arg-1420, Arg-1528, and Ala-1532 (Figure 6D).

Analyzing the three-dimensional alignment between the PCA3p1 peptide and the ARHGAP21 protein (Figure 6E), the peptide PCA3p1 mimicked an ARHGAP21 site that interacts with the *PCA3* promoter. Both docking solutions (PCA3p1-*PCA3*prom and ARHGAP21-*PCA3*prom) pointed to the same binding region on the promoter sequence (between C-192 and C-221), suggesting this range as the most favorable binding site, as demonstrated in Figure 6B and D. The amino acids of the peptide that binds to the promoter corresponds to the same region of ARHGAP21 that binds to the promoter sequence of the *PCA3* gene, although they are not the same residues (Figure 6F).

## Discussion

*PCA3* is a long non-coding RNA that is well characterized as a PCa biomarker detected in tissue and, most importantly, in noninvasive samples (7). In our research, we identified *PCA3* transcripts in the peripheral blood from patients with Pca. Despite not being identified in all patients with PCa, no amplification was verified in BPH samples, showing the high specificity of *PCA3*. Our results are aligned with previous studies (14,25) and demonstrated the effectiveness of *PCA3* as a useful biomarker for PCa discrimination. Although different investigations have been dedicated to the characterization of *PCA3* as a diagnostic and/or prognostic marker, little is known about the regulatory mechanisms controlling its expression.

Herein, we sought to identify recombinant peptides that guided us in characterizing the molecular functioning of the *PCA3* gene. For this purpose, Phage Display technology was performed to select peptides that bind to the promoter sequence of *PCA3*, revealing possible proteins capable of regulating its transcriptional mechanism. We made efforts to validate PCA3p1, the peptide with highest frequency (80%) with great affinity for the *PCA3*prom and similar to ARHGAP21. ARHGAP21 is a member of the RhoGAP family that catalyzes the conversion of active GTP-bound forms of Rho-family GTPases to their inactive GDP-bound state (26). ARHGAP21 has been described to present GAP activity for RhoA and RhoC in PCa cells. However, the activity of ARHGAP21 exerted on RhoA or RhoC may be cell-type specific with particular functions (27,28). In our qPCR assay, basal transcriptional levels of *PCA3* were detected only in LNCaP cells. However, we verified that ARHGAP21 was upregulated in PC-3 cells. A previous work described that the mutation of an A + T-rich region within the *PCA3* promoter is responsible for reduced transcription rate (24) and, as already published by Lazarini et al. (29), ARHGAP21 displays different functions according to prostate cell type. We hypothesized that for PCa progression to a castration-resistant phenotype, a stage represented by the PC-3 cell line, mutation within the *PCA3* promoter inhibits ARHGAP21 recognition, signalizing through a different PCA3-independent pathway, which may be by RhoC (29). In fact, LNCaP cells represent an initial androgen-responsive stage, which is well accepted for being characterized by a high level of *PCA3* expression. This finding likely implies the dependency of these cells to *PCA3*-related mechanisms of growth and cancer progression. Moreover, although the mRNA levels of ARHGAP21 were lower in LNCaP cells, the binding of the *PCA3* promoter region by ARHGAP21 was confirmed through Chip assay suggesting the unique role of ARHGAP21 in PCa cells that express *PCA3*, modulating its molecular functioning.

*PCA3* is located within intron 6 (about 110 kb) of *PRUNE2* gene in the opposite orientation and binds to *PRUNE2* pre-miRNA, acting as a dominant-negative oncogene, down-regulating *PRUNE2* tumor suppressor gene (11). *PRUNE2* contains the downstream BCH coding domain, which has been described to inhibit the function of RhoA. Therefore, *PRUNE2* prevents oncogenic transformation of prostate cells and may control tumor growth through RhoA downregulation (30). We silenced ARHGAP21 in LNCaP cells and quantified *PCA3* and *PRUNE2* transcripts, hypothesizing that, given the ability of *PCA3* to edit *PRUNE2* transcripts, its expression would also be altered in LNCaP-ARHGAP21 silenced cells. As expected, the transcriptional levels of *PCA3* were downregulated and *PRUNE2* was upregulated, which agrees with other studies that report the inverse relationship between *PCA3* and *PRUNE2* (11). When LNCaP cells were stimulated with PCA3p1 phage, there was an increase in *PCA3* expression, a finding that corroborated with the role of ARHGAP21 as a regulatory binding protein of the *PCA3*prom region. However, the treatment increased *PRUNE2* expression. We believe that this effect occurred due to negative feedback, in response to the increase in *PCA3* transcripts, which downregulates *PRUNE2*.

*PCA3* is responsive to androgen stimulation modulating the expression of AR target genes (14). *AR* transcripts were also monitored in LNCaP-ARHGAP21 silenced cells, in which there was a decrease in receptor expression. In LNCaP cells stimulated with PCA3p1 phage, although no significant alterations were observed, there was a tendency of increasing *AR* expression. We demonstrated that silencing ARHGAP21 or treating cells with the ARHGAP21 mimetic peptide led to alterations in the transcription of *PCA3*. Therefore, the obtained results of *AR* expression are in agreement with the activity of *PCA3* in promoting *AR* expression previously observed (14) and confirmed the essential role of ARHGAP21 in enhancing *PCA3* expression.

Regarding docking analyses, ARHGAP21 has three characterized domains, namely the PH, PDZ, and RhoGAP domains. The PH domain, located between amino acids 933-1042, is mainly reported in cell signaling mechanisms, interacting with serine/threonine kinase, G-protein-associated regulators, aptamers, tyrosine kinases, endocytic GTPases, and molecules related with the cytoskeleton (31). The PDZ domain comprises amino acids 48-155aa and is known for its ability to form multimeric protein complexes for binding to the C-terminal portion of different proteins, specifically to the peptide motif (Thr/Ser- X-Val) (32). In addition, this PDZ domain recruits GEF (guanine nucleotide exchange factor) and GAP (GTPase-activating proteins) for adhesion sites (33). The RhoGAP domain names this family of proteins, being responsible for the catalytic activity of converting Rho proteins via GTP-GDP activation, and is located between amino acids 1178 and 1373 (34).

The binding region of PCA3p1 in the promoter region was not concentrated in previously described polymorphism sites, such as TAAA rich sequences, and analyzing the conserved domains of ARHGAP21 and the alignment site with the peptide PCA3p1 (near 1350-1400aa), we believe that the binding of ARHGAP21 in the *PCA3*prom segment can somehow prevent the interaction of ARHGAP21 with RhoGTPase through a competition for its binding site. It is important to note that in the western blot assay, different silenced bands were noted, suggesting the presence of protein isoforms (35). However, the region of ARHGAP21 mimicked by the peptide PCA3p1 was found within the consensus sequence between the isoforms, as also pointed out in our *in silico* analyses. Although the amino acids in PCA3p1 and ARHGAP21 responsible for binding through polar interaction with *PCA3* promoter sequence were different, the interaction sites were within the same region. This was expected, as the three-dimensional structure and adjacent residues of each structure are different.

In summary, we identified, for the first time, a role of ARHGAP21 in binding to the promoter region of *PCA3* and in regulating *PCA3* expression. Since *PCA3* is involved in PCa malignancy, our results contribute significantly to a better knowledge of *PCA3* transcriptional regulation and could support the development of new therapeutic strategies.

## References

[1] 1. de Wit R, Freedland SJ, Oudard S, Marinov G, Capart P, Combest AJ, et al. Real-world evidence of patients with metastatic castration-resistant prostate cancer treated with cabazitaxel: comparison with the randomized clinical study CARD. Prostate Cancer Prostatic Dis 2022; 26: 67-73, doi: 10.1038/s41391-021-00487-1. 10.1038/s41391-021-00487-1 PMC10023563 35039605

[2] 2. Carlsson SV, Vickers AJ. Screening for prostate cancer. Med Clin North Am 2020; 104: 1051-1062, doi: 10.1016/j.mcna.2020.08.007. 10.1016/j.mcna.2020.08.007 PMC8287565 33099450

[3] 3. Bussemakers MJG, Van Bokhoven A, Verhaegh GW, Smit FP, Karthaus HFM, Schalken JA, et al. DD3: a new prostate-specific gene, highly overexpressed in prostate cancer. Cancer Res 1999; 59: 5975-5979. 10606244

[4] 4. Kim JH, Hong SK. Clinical utility of current biomarkers for prostate cancer detection. Investig Clin Urol 2021; 62: 1-13, doi: 10.4111/icu.20200395. 10.4111/icu.20200395 PMC7801171 33381926

[5] 5. Ploussard G, de la Taille A. The role of prostate cancer antigen 3 (PCA3) in prostate cancer detection. Exp Rev Anticancer Ther 2018; 18: 1013-1020, doi: 10.1080/14737140.2018.1502086. 10.1080/14737140.2018.1502086 30016891

[6] 6. Dijkstra S, Mulders PFA, Schalken JA. Clinical use of novel urine and blood based prostate cancer biomarkers: a review. Clin Biochem 2014; 47: 889-896, doi: 10.1016/j.clinbiochem.2013.10.023. 10.1016/j.clinbiochem.2013.10.023 24177197

[B07] 7. Neves AF, Dias-Oliveira JDD, Araújo TG, Marangoni K, Goulart LR. Prostate cancer antigen 3 (PCA3) RNA detection in blood and tissue samples for prostate cancer diagnosis. Clin Chem Lab Med 2013; 51: 881-887, doi: 10.1515/cclm-2012-0392. 10.1515/cclm-2012-0392 23241599

[8] 8. Wang K, Zhao H, Wang W, Zhu Y, Zhang X, Ma J, et al. Effect of upregulation of DD3 on early detection and prognosis in prostate cancer. Transl Androl Urol 2020; 9: 1550-1558, doi: 10.21037/tau-19-899. 10.21037/tau-19-899 PMC7475679 32944517

[9] 9. Marangoni K, Neves AF, Rocha RM, Faria PR, Alves PT, Souza AG, et al. Prostate-specific RNA aptamer: promising nucleic acid antibody-like cancer detection. Sci Rep 2015; 5: 12090, doi: 10.1038/srep12090. 10.1038/srep12090 PMC4502603 26174796

[10] 10. Neves AF, Araújo TG, Biase WKFS, Meola J, Alcântara TM, Freitas DG, et al. Combined analysis of multiple mRNA markers by RT-PCR assay for prostate cancer diagnosis. Clin Biochem 2008; 41: 1191-1198, doi: 10.1016/j.clinbiochem.2008.06.013. 10.1016/j.clinbiochem.2008.06.013 18640109

[11] 11. Salameh A, Lee AK, Cardó-Vila M, Nunes DN, Efstathiou E, Staquicini FI, et al. PRUNE2 is a human prostate cancer suppressor regulated by the intronic long noncoding RNA PCA3. Proc Natl Acad Sci USA 2015; 112: 8403-8408, doi: 10.1073/pnas.1507882112. 10.1073/pnas.1507882112 PMC4500257 26080435

[12] 12. Schalken JA, Hessels D, Verhaegh G. New targets for therapy in prostate cancer: differential display code 3 (DD3PCA3), a highly prostate cancer-specific gene. Urology 2003; 62 (5 Suppl 1): 34-43, doi: 10.1016/S0090-4295(03)00759-3. 10.1016/s0090-4295(03)00759-3 14607216

[13] 13. Zhou W, Chen Z, Hu W, Shen M, Zhang X, Li C, et al. Association of short tandem repeat polymorphism in the promoter of prostate cancer antigen 3 gene with the risk of prostate cancer. PLoS One 2011; 6: e20378, doi: 10.1371/journal.pone.0020378. 10.1371/journal.pone.0020378 PMC3105025 21655300

[14] 14. Ferreira LB, Palumbo A, de Mello KD, Sternberg C, Caetano MS, de Oliveira FL, et al. PCA3 noncoding RNA is involved in the control of prostate-cancer cell survival and modulates androgen receptor signaling. BMC Cancer 2012; 12: 507, doi: 10.1186/1471-2407-12-507. 10.1186/1471-2407-12-507 PMC3544699 23130941

[15] 15. Lemos AEG, Ferreira LB, Batoreu NM, de Freitas PP, Bonamino MH, Gimba ERP. PCA3 long noncoding RNA modulates the expression of key cancer-related genes in LNCaP prostate cancer cells. Tumor Biol 2016; 37: 11339-11348, doi: 10.1007/s13277-016-5012-3. 10.1007/s13277-016-5012-3 26960690

[16] 16. Lázaro-Silva DN, De Mattos JCP, Castro HC, Alves GG, Amorim LMF. The use of DNA extraction for molecular biology and biotechnology training: a practical and alternative approach. Creat Educ 2015; 06: 762-772, doi: 10.4236/ce.2015.68079.

[17] 17. Rio DC, Ares Jr M, Hannon GJ, Nilsen TW. Purification of RNA using TRIzol (TRI Reagent). Cold Spring Harb Protoc 2010; 2010: pdb.prot5439, doi: 10.1101/pdb.prot5439. 10.1101/pdb.prot5439 20516177

[18] 18. Barbas CF et al. Phage Display: a laboratory manual. Cold Spring Harbor Laboratory Press. 2001.

[19] 19. Mota STS, Vecchi L, Alves DA, Cordeiro AO, Guimarães GS, Campos-Fernández E, et al. Annexin A1 promotes the nuclear localization of the epidermal growth factor receptor in castration-resistant prostate cancer. Int J Biochem Cell Biol 2020; 127: 105838, doi: 10.1016/j.biocel.2020.105838. 10.1016/j.biocel.2020.105838 32858191

[20] 20. Yang J, Zhang Y. I-TASSER server: new development for protein structure and function predictions. Nucleic Acids Res 2015; 43: W174-W181, doi: 10.1093/nar/gkv342. 10.1093/nar/gkv342 PMC4489253 25883148

[21] 21. Lamiable A, Thévenet P, Rey J, Vavrusa M, Derreumaux P, Tufféry P. PEP-FOLD3: faster de novo structure prediction for linear peptides in solution and in complex. Nucleic Acids Res 2016; 44: W449-W454, doi: 10.1093/nar/gkw329. 10.1093/nar/gkw329 PMC4987898 27131374

[22] 22. Pontius J, Richelle J, Wodak SJ. Deviations from standard atomic volumes as a quality measure for protein crystal structures. J Mol Biol 1996; 264: 121-136, doi: 10.1006/jmbi.1996.0628. 10.1006/jmbi.1996.0628 8950272

[23] 23. Anderson RJ, Weng Z, Campbell RK, Jiang X. Main-chain conformational tendencies of amino acids. Proteins 2005; 60: 679-689, doi: 10.1002/prot.20530. 10.1002/prot.20530 16021632

[24] 24. Verhaegh GW, van Bokhoven A, Smit F, Schalken JA, Bussemakers MJG. Isolation and characterization of the promoter of the human prostate cancer-specific DD3 gene. J Biol Chem 2000; 275: 37496-37503, doi: 10.1074/jbc.M006293200. 10.1074/jbc.M006293200 10982808

[25] 25. McKillop C. Interview with Jack Schalken. PCA3 and its use as a diagnostic test in prostate cancer. Interview by Christine. Eur Urol 2006; 50: 153-154, doi: 10.1016/j.eururo.2006.04.021; PMID: 16713069. 10.1016/j.eururo.2006.04.021 16713069

[26] 26. Etienne-Manneville S, Hall A. Rho GTPases in cell biology. Nature 2002; 420: 629-635, doi: 10.1038/nature01148. 10.1038/nature01148 12478284

[27] 27. Li Y, Zeng B, Li Y, Zhang C, Ren G. Downregulated expression of ARHGAP10 correlates with advanced stage and high Ki-67 index in breast cancer. PeerJ 2019; 2019: e7431, doi: 10.7717/peerj.7431. 10.7717/peerj.7431 PMC6679923 31396458

[28] 28. Schmidt A, Hall A. Guanine nucleotide exchange factors for Rho GTPases: turning on the switch. Genes Dev 2002; 16: 1587-1609, doi: 10.1101/gad.1003302. 10.1101/gad.1003302 12101119

[29] 29. Lazarini M, Traina F, Machado-Neto JA, Barcellos KSA, Moreira YB, Brandão MM, et al. ARHGAP21 is a RhoGAP for RhoA and RhoC with a role in proliferation and migration of prostate adenocarcinoma cells. Biochim Biophys Acta Mol Basis Dis 2013; 1832: 365-374, doi: 10.1016/j.bbadis.2012.11.010. 10.1016/j.bbadis.2012.11.010 23200924

[30] 30. Clarke RA, Zhao Z, Guo AY, Roper K, Teng L, Fang ZM, et al. New genomic structure for prostate cancer specific gene PCA3 within BMCC1: Implications for prostate cancer detection and progression. PLoS One 2009; 4: e4995, doi: 10.1371/journal.pone.0004995. 10.1371/journal.pone.0004995 PMC2655648 19319183

[31] 31. Ménétrey J, Perderiset M, Cicolari J, Dubois T, Elkhatib N, El Khadali F, et al. Structural basis for ARF1-mediated recruitment of ARHGAP21 to Golgi membranes. EMBO J 2007; 26: 1953-1962, doi: 10.1038/sj.emboj.7601634. 10.1038/sj.emboj.7601634 PMC1847662 17347647

[32] 32. Doyle DA, Lee A, Lewis J, Kim E, Sheng M, MacKinnon R. Crystal structures of a complexed and peptide-free membrane protein- binding domain: molecular basis of peptide recognition by PDZ. Cell 1996; 85: 1067-1076, doi: 10.1016/S0092-8674(00)81307-0. 10.1016/s0092-8674(00)81307-0 8674113

[33] 33. Rosa LRO, Soares GM, Silveira LR, Boschero AC, Barbosa-Sampaio HCL. ARHGAP21 as a master regulator of multiple cellular processes. J Cell Physiol 2018; 233: 8477-8481, doi: 10.1002/jcp.26829. 10.1002/jcp.26829 29856495

[34] 34. Lu S, Wang J, Chitsaz F, Derbyshire MK, Geer RC, Gonzales NR, et al. CDD/SPARCLE: the conserved domain database in 2020. Nucleic Acids Res 2020; 48: D265-D268, doi: 10.1093/nar/gkz991. 10.1093/nar/gkz991 PMC6943070 31777944

[35] 35. Carles A, Millon R, Cromer A, Ganguli G, Lemaire F, Young J, et al. Head and neck squamous cell carcinoma transcriptome analysis by comprehensive validated differential display. Oncogene 2006; 25: 1821-1831, doi: 10.1038/sj.onc.1209203. 10.1038/sj.onc.1209203 16261155

